# Gastrointestinal symptoms as first remarkable signs of ANCA-associated granulomatosis with polyangiitis: a case report and reviews

**DOI:** 10.1186/s12876-021-01730-8

**Published:** 2021-04-08

**Authors:** Nóra Ledó, Ákos Géza Pethő

**Affiliations:** Department of Internal Medicine and Oncology, Faculty of Medicine, Semmelweis University, Budapest, Hungary

**Keywords:** Antineutrophil cytoplasmic antibodies, Systemic vasculitis, Granulomatosis with polyangiitis, Gastrointestinal haemorrhage, Case report

## Abstract

**Background:**

Systemic vasculitis associated with antineutrophil cytoplasmic autoantibodies (ANCA) have an extremely wide variety of symptoms, therefore the fast and proper diagnosis is difficult to establish even for experienced physicians. Gastrointestinal manifestations in ANCA-associated granulomatosis with polyangiitis (GPA) may be present, however, severe, life-threatening complications (such as perforations) are rare.

**Case presentation:**

A case of an 18-year-old male patient is presented, where gastrointestinal symptoms (abdominal pain, vomiting, diarrhoea) were the first remarkable signs of GPA. The initial diagnosis of inflammatory bowel disease delayed the administration of proper immunosuppressive therapy, which might have contributed to the rare and life-threatening complication of arterial duodenal bleeding with perforation. Our systematic review of the literature found only a few case reports where gastrointestinal symptoms were the first signs of GPA, however, this entity might be more frequent if physicians would think of this possibility more often.

**Conclusions:**

Gastrointestinal bleeding is a rare but potential lethal complication of vasculitis. Consequently, we recommend investigating the patients diagnosed with GPA for gastrointestinal bleeding during the treatment.

## Background

Systemic vasculitis can produce a wide variety of clinical manifestations depending on the localization of the affected vessels, and often recognized only when severe conditions and rapid progression are present.

Systemic vasculitis is classified based on the size of the inflamed vessels, as large-vessel vasculitis, medium-vessel vasculitis, small-vessel vasculitis and variable-vessel vasculitis. Small-vessel vasculitis can be further divided into immune complex-mediated and ANCA-associated small-vessel vasculitis. ANCA-associated vasculitis is a necrotizing vasculitis with a few or no immune deposits in vessel walls (called pauci-immune), and usually associated with circulating antineutrophil cytoplasmic autoantibodies (ANCAs), however ANCA positivity is not mandatory. Based on the clinical and pathological findings, there are three types of ANCA-associated small-vessel vasculitis: microscopic polyangiitis (MPA), granulomatosis with polyangiitis (GPA, Wegener’s granulomatosis) and eosinophilic granulomatosis with polyangiitis (EGPA, Churg-Strauss syndrome) [1]. Renal manifestations are common in MPA and GPA, while symptoms of the lung and respiratory tract are more frequent in EGPA and GPA. In GPA, ears, nose and throat are usually affected. Cutaneous, musculoskeletal, neurological and gastrointestinal (GI) manifestations may be also present but usually not characteristic [1, 2].

Because of the wide variety of the symptoms, ANCA-associated vasculitis is challenging to be recognized even for experienced physicians. Delay of the adequate immunosuppressive therapy can accelerate the progression of the disease. Here we present the case of a young male patient with ANCA-associated vasculitis, where the first remarkable signs of the disease were gastrointestinal symptoms. The unusual manifestation led to a delayed diagnosis and therapy, which could contribute to a life-threatening complication. There are previous reports about GI symptoms of the disease, presented the cases as very rare entities. Based on our experience from a nephrology department, it is still very important to emphasise these unusual manifestations of ANCA-associated vasculitis, because they are not as rare as thought. Recognizing the disease in early stage can save lives.

## Case presentation

An 18-year-old, previously healthy white male patient was admitted to our Nephrology Department with elevated serum creatinine levels. His symptoms started 2 weeks ago with severe abdominal pain, vomiting, hematemesis, and diarrhoea and was examined by two other hospitals. At the first hospital, CT (computer tomography) scan showed ileal wall thickening. The patient declined to undergo colonoscopy, therefore based on the result of the CT scan, laboratory tests (C-reactive protein 149 mg/L) and the symptoms, inflammatory bowel disease (IBD) was diagnosed. The recommended therapy was sulfasalazine, methylprednisolone, and metronidazole. At his first symptoms, his kidney function was normal with a 90 µmol/L serum creatinine level and a 5.9 mmol/L BUN (blood urea nitrogen) level, although microhaematuria was detected (no sediment evaluation). His abdominal pain was worsened, so 10 days later, he was admitted to the second hospital, where the total number of the white blood cells (21.3 × 10^9^/L), C-reactive protein (137 mg/L) and serum creatinine level (198 μmol/L) found elevated. Blood was not detected in the stool but calprotectin level, one of the potential indicators of IBD, was elevated (776 µg/g). The patient’s renal function started to decline rapidly reaching a serum creatinine level of 429 μmol/L in 5 days. Microscopic examination of the urinary sediment showed dysmorphic red blood cells. He developed anaemia with a haemoglobin level of 105 g/L. Abdominal ultrasound examination could not find any bowel wall thickening but found hyperechoic enlarged kidneys. The patient was transferred to our Nephrology Department for further diagnostics.

**Fig. 1:**
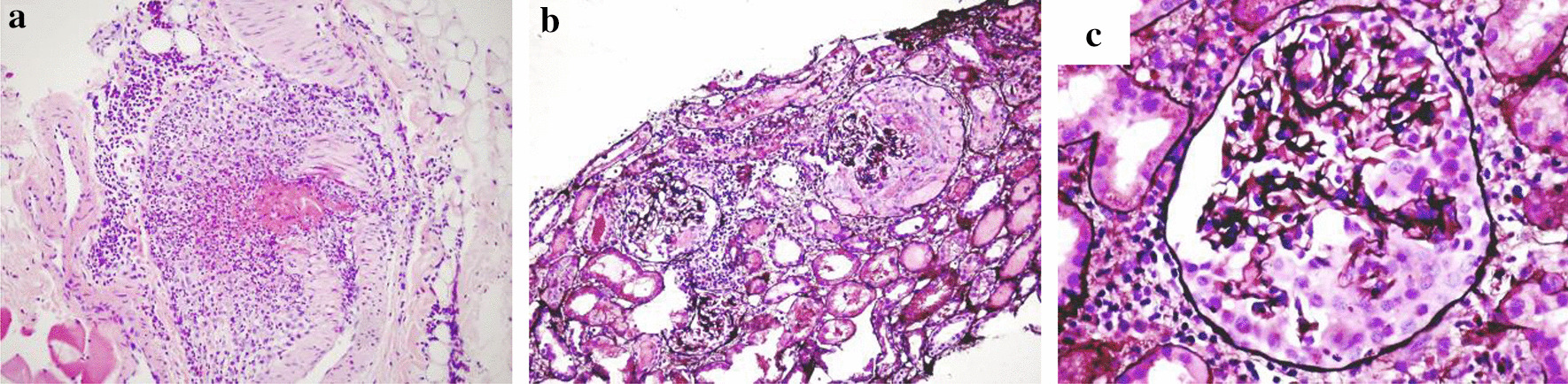
Signs of small-vessel vasculitis and crescentic glomerulonephritis in the patient’s kidney biopsy sample. Inflammation of a small arteriole close to the renal capsule with fibrinoid necrosis. Magnification: × 200, haematoxylin–eosin (**a**). Two affected glomeruli with fibrinoid necrosis and crescent formation. Magnification: × 200, Jones’ stain (**b**). Crescent formation in a glomerulus. Magnification: × 600, Jones’ stain (**c**)

At the Nephrology Department, anti-proteinase 3 ANCA (PR3-ANCA) positivity (461 U) was detected, and urgent kidney biopsy showed pauci-immune, crescentic glomerulonephritis with the signs of vasculitis (Fig. 1). Based on these results, ANCA-associated vasculitis with rapidly progressive glomerulonephritis was diagnosed, and high dose intravenous (IV) methylprednisolone (500 mg/day for 3 days followed by slow dose reduction), IV cyclophosphamide (500 mg) and plasma exchange therapy were commenced with continuing the previously administered anti-hypertensive and proton-pump inhibitor therapy. Trimethoprim/sulfamethoxazole therapy was started according to the recommendations [3]. Because of the progressive renal function decline, haemodialysis therapy was started on the fifth day of admission.

The patient had a 67 g/L of haemoglobin level on the fifth day of admission; therefore, blood transfusion was performed. In search of the cause of his anaemia, we performed examinations to exclude extrarenal bleeding. Several stool samples were tested for blood, which were mostly negative, with one slightly positive sample (regular Weber test). An upper gastrointestinal endoscopy was performed, which showed normal oesophagus, stomach and duodenum without any sings of bleeding or inflammation. Clinically, the patient did not have bleeding from the respiratory tract. Chest X-ray and high-resolution CT scan were performed, which showed small nodules and ground glass opacity signs in a small area of the right lower lobule of the lung. Examination at the otorhinolaryngology department found some ulcers on the nasal septum. Haemolysis was also excluded, and erythropoietin and Vitamin B12 injections were performed because of the renal disease and proven Vitamin B12 deficiency.

Based on the criteria of vasculitis [4] (clinical symptoms, nasal ulceration, necrotising glomerulonephritis, elevated PR3-ANCA levels), GPA was diagnosed. The patient was discharged on the 24th day after his admission, after his second administration of IV cyclophosphamide.

Three days later, the patient was admitted to our hospital because of an early morning haematochezia with severe fatigue. He had epigastric abdominal pain the day before. With physical examination, the patient was pale with a new systolic murmur, with a mild epigastric and umbilical tenderness. The urgent laboratory test showed severe anaemia with a haemoglobin level of 36 g/L. With continuing haematochezia and haematemesis, the patient presented in a rapidly progressive haemorrhagic shock. Proton-pump inhibitor therapy started IV immediately. To stabilize the patient, 4500 ml crystalloid solution, several transfusions (17 units of red blood cell concentrates, 4 units of fresh frozen plasma and 24 units of platelet concentrates) were needed during the day and tranexamic acid was also administered. Gastroscopy was performed, during which “spurting” arterial bleeding of the duodenum was found. The bleeding was controlled with three clips placed on the artery. The patient stayed conscious and normotensive, the haemoglobin level could be increased to 67 g/L, and for further observation, he was transferred to the intensive care unit of our hospital.

**Fig. 2:**
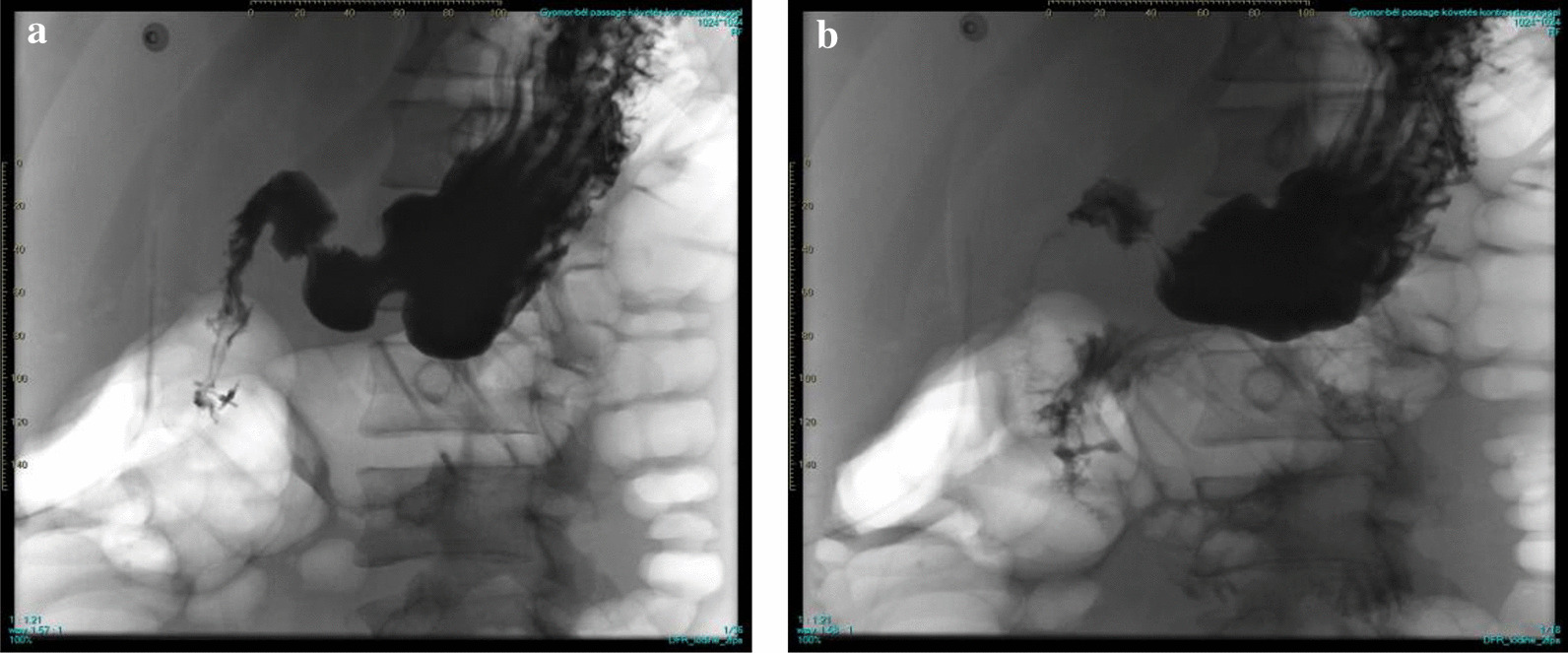
Contrast radiography of the upper gastrointestinal tract 3 days after the surgery. Early contrast radiography of the stomach (**a**). Late contrast radiography with contrast agent diffusion next to the clipped duodenum (**b**)

**Fig. 3:**
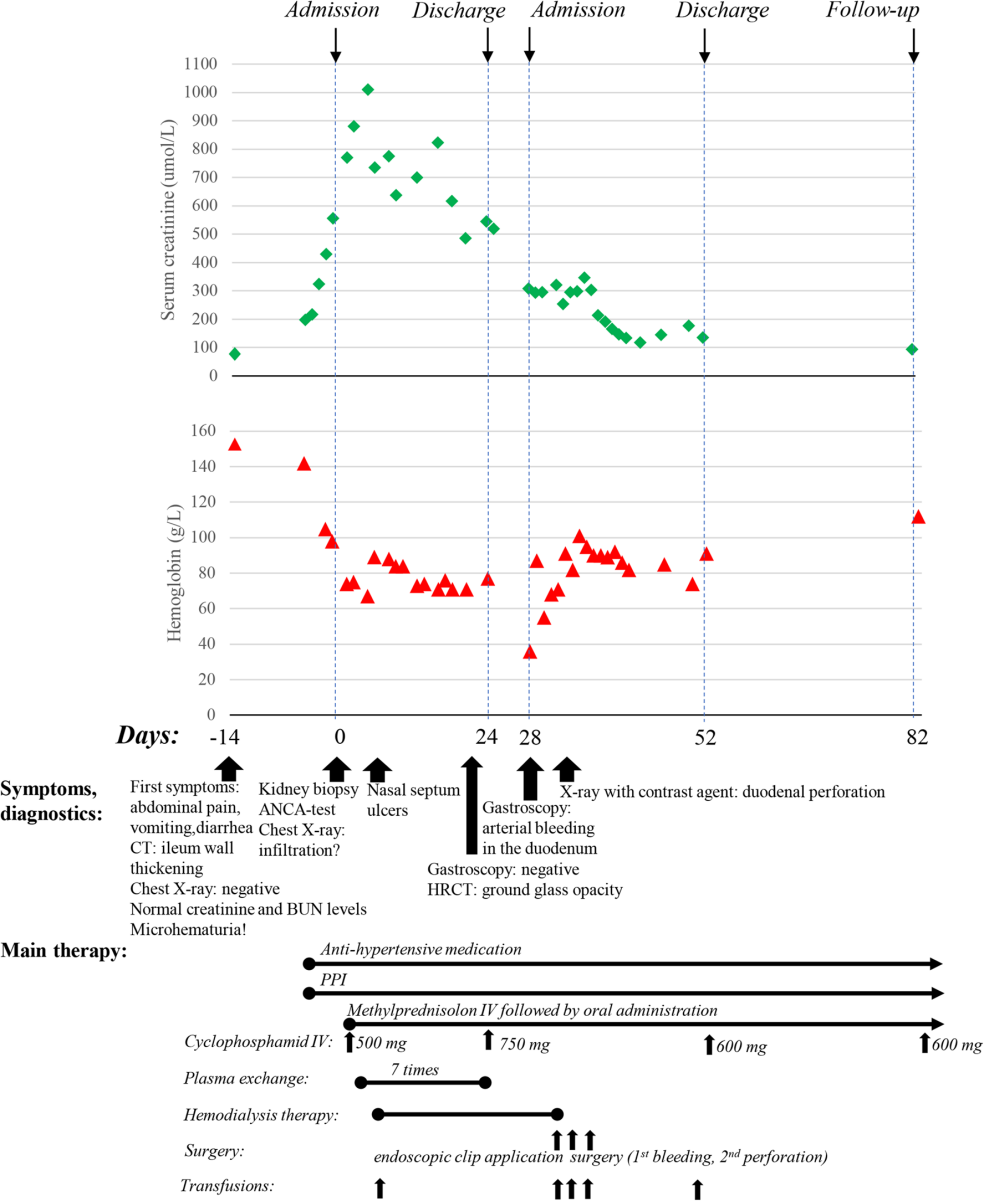
Summary of the main aspects of the reported case. The upper chart shows the serum creatinine levels (μmol/L) during the observation of the patient, while haemoglobin levels (g/L) are presented in the lower chart. The symptoms, diagnostic tests and treatment are summarized in chronologic order. *ANCA* antineutrophil cytoplasmic autoantibodies, *BUN* blood urea nitrogen, *CT* computer tomography, *HRCT* high resolution CT, *PPI* proton-pump inhibitor, *IV* intravenous

At the intensive care unit, 2 days later the bleeding restarted next to the clips, and the patient needed surgery to suture the artery. Three days after the first surgery, a reoperation was needed because of the perforation of the duodenum (Fig. 2). After an additional 10 days of intensive therapy, the patient recovered from the second surgery. Enteral nutrition could be administered, and haemodialysis therapy could be terminated. After this, he was transferred back to our department, and the cyclophosphamide therapy was continued. He left the hospital after 10 days of observation. Figure 3 summarizes the main points of the case description.

## Discussion and conclusions

Granulomatosis with polyangiitis (GPA) may have gastrointestinal manifestations, such as ulcers, bleeding, ischemic bowel disease, peritonitis, ischemic hepatitis. The most common sign of the gastrointestinal manifestation of GPA is abdominal pain [5]. Pagnoux et al. [5] found that among patients with systemic small-vessel vasculitis in 22 years, only 62 had gastrointestinal tract involvement (18%), and out of the 62 patients, only 6 were diagnosed with GPA. GI disease was found in 7% of 673 patients with MPA or GPA included in the cluster analysis of the French Vasculitis Study Group and the European Vasculitis Society [6]. In a recent study from Sweden found that only 6% of the 216 patients with GPA or MPA observed in a single centre had gastrointestinal involvement [7]. Abdominal pain and GI bleeding were the most common GI symptoms in the database and only 5 patients (2%) had intestinal perforation as the complication of the disease. The same criteria were used as in the previous study [5]: (a) GI symptoms, such as diffuse abdominal pain with acute onset or GI bleeding, that were present at the time of GPA or MPA diagnosis (or within the next 3 months) and responded to specific therapy for vasculitis; (b) GI symptoms that occurred during a relapse, diagnosed on the basis of extraintestinal features of GPA or MPA and/or responded to specific therapy for vasculitis; (c) and/or GI tract vasculitis that was histologically proven on biopsy or at autopsy were taken into consideration [7].

Gastrointestinal perforation is also a rare but severe complication of ANCA-associated vasculitis. A case report presented a patient with known GPA who suffered multiple ileal perforations, and the review of the literature showed, that 13 similar cases were presented previously [8]. A Turkish case report also presented a female patient where intestinal perforation was the first main sign of GPA [9].

There are a few case reports in the literature where GI symptoms are the leading manifestation in GPA, however usually there were other, mild symptoms previously present which were not taken into consideration promptly. Skaife et al. [10] presented a case, where intestinal perforations were the initial signs of GPA, however respiratory and renal involvement were also present. Steele et al. [11] presented a case in 2001 where upper gastrointestinal bleeding and colitis were the main manifestation of GPA, but the patient’s disease started with joint pain. Also, a case report describes upper gastrointestinal bleeding with epigastric abdominal pain and hematemesis as the first main symptoms of GPA, however the patient had recurrent sinusitis before the GI manifestation [12]. A 32-old man had severe intestinal ulceration, where GPA was proved based on the symptoms of recurrent fever, nasal bleeding, haematuria, and proteinuria. The ulcerations disappeared after the combination therapy of prednisolone and cyclophosphamide [13]. A 70-year-old female patient had severe haematochezia due to GPA; however, the main symptoms were related to the respiratory system: chronic productive cough for 6 months and respiratory failure at the time of admission [14].

Additionally, in the case report of Shahedi et al. showed that GPA could mimic inflammatory bowel disease, as the chronic diarrhoea and haematochezia lasted more than 1 year before the correct diagnosis of GPA, which was proved with kidney biopsy and elevated PR3-ANCA titre. Kidney function decline was the clue in making the right diagnosis [15]. A case report from Poland also revealed the difficulties of the proper diagnosis when GPA and IBD mimic each other’s symptoms. A patient first was diagnosed with Crohn’s disease, while GPA was discovered later due to the fever, joint pain, rash, respiratory tract and kidney manifestations [16]. It is also important to remember, that fecal calprotectin level can be elevated in different types of vasculitis with GI involvement, such as Behçet disease and IgA-vasculitis [17, 18]. Serum calprotectin level was also described as a biomarker of ANCA-associated glomerulonephritis to predict disease activity and relapse rate [19, 20]. Neutrophils of GPA patients trigger the induction of S100A9 (part of calprotectin), which contribute to the tissue-invasive capability of the disease [21]. Based on these results, fecal calprotectin might be a good biomarker to show the severity of GI involvement in ANCA-associated vasculitis.

Although, the possible gastrointestinal manifestations are known in GPA, there are only a few cases available in the medical literature when the gastrointestinal symptoms were the first and only sings of the disease (details shown in Table 1) [22–24]. Our case is the fourth reported in the available medical literature, where GI manifestation was the first and only remarkable sign of GPA.

**Table 1:** Summary of case reports in English language literature, where gastrointestinal manifestations were reported as first signs of granulomatosis with polyangiitis (GPA)

References	Patient’s age	Sex	First manifestation of GPA	Other manifestations at the beginning	Other manifestations during the disease
Qian et al. [22]	79	Female	Haemorrhagic pancolitis	No	Renal and respiratory involvement
Sinnoth et al. [23]	29	Male	Haemorrhagic colitis	No	Renal and respiratory involvement
Yoshikawa et al. [24]	30	Male	Oral aphtha, mucosal bleeding in the colon	No	Skin ulcer, lesion of the paranasal sinus
Current case	18	Male	Abdominal pain, vomiting, diarrhoea	No	Renal involvement, mild respiratory involvement, arterial bleeding in the duodenum with perforation

Here we presented a case, where gastrointestinal symptoms were the first remarkable signs of GPA, other manifestations (such as progressive glomerulonephritis) started later. After the correct diagnosis of the ANCA-associated vasculitis, we reviewed the patient’s previous documentation again, where we found documented dipstick urinalysis with microscopic haematuria in one of his first examined urine samples. The lack of microscopic urinary sediment examination leaves the question unanswered: whether those erythrocytes were dysmorphic and signs of glomerular disease or not. We hypothesize that the initial GI symptoms (bloody diarrhoea and abdominal pain) were signs of GI involvement of GPA and the delayed administration of the proper immunosuppressive therapy might have contributed to the rare life-threatening complication seen in this patient. This explanation supported by the gastroscopy performed 10 days before the duodenal bleeding, where no mucosal alterations or bleeding were found. At the urgent gastroscopy during the bleeding, similar, normal mucosa was found, except the arterial, described as ‘spurting’ bleeding in the duodenum. Histologic evidence would be the best to prove the underlying cause, but in these clinical situations, GI biopsy could not be performed.

In the reviewed literature we found only a few cases where GI symptoms were the first signs of GPA [22, 24]; however it must be present more often, as previous studies showed [5–7]]. Therefore, we suggest that the differential diagnostics of GI bleeding should always consider ANCA-associated vasculitis as a rare but serious condition. The proper microscopic examination of urinary sediment is a non-invasive and important tool in the diagnostics of kidney diseases, which in the case of (micro)haematuria should be performed. Additionally, gastrointestinal bleeding is a rare but potential lethal complication of vasculitis. Consequently, we recommend investigating the patients diagnosed with GPA for gastrointestinal bleeding during the treatment, especially if there were gastrointestinal symptoms among the first clinical signs of the vasculitis. This forced investigation for gastrointestinal bleeding could prevent the patients from potentially lethal complication of vasculitis.

## Data Availability

Data sharing is not applicable to this article as no datasets were generated or analysed during the current study.

## Abbreviations

ANCA: Antineutrophil cytoplasmic autoantibodies
BUN: Blood urea nitrogen
CT: Computer tomography
EGPA: Eosinophilic granulomatosis with polyangiitis
GI: Gastrointestinal
GPA: Granulomatosis with polyangiitis
HRCT: High resolution CT
IBD: Inflammatory bowel disease
IV: Intravenous
MPA: Microscopic polyangiitis
PPI: Proton-pump inhibitor
PR3-ANCA: Anti-proteinase 3 ANCA
